# Surgical Outcomes of 2041 Consecutive Laparoscopic Gastrectomy Procedures for Gastric Cancer: A Large-Scale Case Control Study

**DOI:** 10.1371/journal.pone.0114948

**Published:** 2015-02-02

**Authors:** Jian-Xian Lin, Chang-Ming Huang, Chao-Hui Zheng, Ping Li, Jian-Wei Xie, Jia-Bin Wang, Jun Lu, Qi-Yue Chen, Long-Long Cao, Mi Lin

**Affiliations:** Department of Gastric Surgery, Fujian Medical University Union Hospital, No.29 Xinquan Road, Fuzhou 350001, Fujian Province, China; Gastroenterological Surgery, JAPAN

## Abstract

**Background:**

Laparoscopic gastrectomy (LG) for gastric cancer has increased in popularity due to advances in surgical techniques. The aim of this study is to validate the efficacy and safety of laparoscopic gastrectomy for gastric cancer compared with open gastrectomy (OG).

**Methods:**

The study comprised 3,580 patients who were treated with curative intent either by laparoscopic gastrectomy (2,041 patients) or open gastrectomy (1,539 patents) between January 2005 and October 2013. The surgical outcomes were compared between the two groups.

**Results:**

Laparoscopic gastrectomy was associated with significantly less blood loss, transfused patient number, time to ground activities, and post-operative hospital stay, but with similar operation time, time to first flatus, and time to resumption of diet, compared with the open gastrectomy. No significant difference in the number of lymph nodes dissected was observed between these two groups. The morbidity and mortality rates of the LG group were comparable to those of the OG group (13.6% vs. 14.4%, P = 0.526, and 0.3% vs. 0.2%, P = 0.740). The 3-year disease-free and overall survival rates between the two groups were statistically significant (P<0.05). According to the UICC TNM classification of gastric cancer, the 3-year disease-free and overall survival rates were not statistically different at each stage.

**Conclusions:**

Our single-center study of a large patient series revealed that LG for gastric cancer yields comparable surgical outcomes. This result was also true of local advanced gastric cancer (AGC). A well-designed randomized controlled trial comparing surgical outcomes between LG and OG in a larger number of patients for AGC can be carried out.

## Introduction

Currently, surgical resection using gastrectomy and proper perigastric lymphadenectomy is the only treatment option to enhance the survival rate of patients with gastric cancer [1]. Since the use of laparoscopic techniques for early gastric cancer was first reported in 1994 [2], laparoscopic gastrectomy (LG) for gastric cancer has become popular due to the many advantages of minimally invasive surgery and advances in surgical techniques [3–5]. However, LG is still classified as an investigational treatment in the most recent Japanese Guideline because of the lack of a prospective study with a sufficient sample size sufficient to prove its benefits [6]. On the other hand, in most countries, more than 80% of patients with gastric cancer are diagnosed with advanced gastric cancer (AGC). There is a lack of large-scale data concerning the long-term outcomes for these patients, so the use of LG for the treatment of AGC is still a contentious issue [7, 8]. Therefore, we present the usefulness of laparoscopic gastrectomy for gastric cancer based on our experience with over 2000 cases.

## Patients and Methods

### Patients

Between January 2005 to October 2013, 3580 patients diagnosed with primary gastric cancer were treated with curative resection at the Department of Gastric Surgery, Fujian Medical University Union Hospital, Fuzhou, China. Of these patients, 2041 underwent a laparoscopic approach and 1539 patients underwent an open technique. Patients who diagnosed with cT1 to cT4a and without clinical evidence of extraperigastric lymph nodes and distant metastases were informed of the possible complications of the procedure and the advantages and disadvantages of a laparoscopic compared with an open approach. Written informed consent was obtained from all patients prior to the operation. A retrospective analysis was performed, using a prospectively maintained comprehensive database, to determine the technical pitfalls of the procedure. Patient demographics, underlying diseases, data on surgery, and data on preoperative and postoperative monitoring including complications and length of hospital stay were recorded in a clinical database system [9]. The surgical procedures were described in detail as follows: 1) total or subtotal gastrectomy was performed, according to tumor location, size, and depth of invasion. 2) D1+α (dissection of group 1 and number 7 lymph node), D1+β(dissection of group 1 and number 7, 8a, 9 lymph nodes), or D2 lymphadenectomy (dissection of all group 1 and group 2 lymph nodes) were undertaken according to the rules of the Japanese Research Society for Gastric Cancer [10]. The operation time was measured from the first skin incision to the closure of all skin incisions with skin staples. Staging was determined according to the 7th edition of the International Union against Cancer (UICC) TNM classification [11]. Adjuvant chemotherapy with 5-fluorouracil (5-FU)-based regimens (mostly 5-FU with cisplatin) was recommended to most patients with advanced gastric cancer.

Postoperative follow-ups were performed every 3 months for 2 years, and then every 6 months from 3 to 5 years. Most patients’ routine follow-up appointments included a physical examination, laboratory tests (including CA19-9, CA72-4, and CEA levels), chest radiography, abdominopelvic ultrasonography or computed tomography (CT), and an annual endoscopic examination. If gastrointestinal symptoms were reported, an additional examination was carried out. The survival time was designated as the time from operation until the date that the survival information was collected or the date of death. All patients were observed until death or the final follow-up date of June 2014, whichever occurred first.

### Ethics Statement

Ethics committee of Fujian medical union hospital approved this retrospective study. Written consent was given by the patients for their information to be stored in the hospital database and used for research.

### Statistical analysis

Statistical analysis was performed using SPSS.v16.0 for Windows (SPSS Inc., Chicago, IL). The statistical analysis was conducted by Student’s t test or chi-square test, and cumulative survival was compared by Kaplan—Meier method and log rank test. Values of p <0.05 were considered statistically significant.

## Results

### Clinicopathological Characteristics of Patients

The clinicopathological characteristics of the 3580 patients (2041 laparoscopic vs. 1539 open) are listed in Table 1. There were 2693 males and 887 females whose ages ranged from 12 to 91 years (mean age was 60.5±11.1 years). For patients in the LG group, a distal gastrectomy was performed in 46.0% of patients, a total gastrectomy in 51.4%, and a proximal gastrectomy in 2.6% of patients. Meanwhile, for the OG group, a distal gastrectomy was performed in 42.4% of patients, a total gastrectomy in 54.5%, and a proximal gastrectomy in 3.1% of patients. According to the UICC TNM classification of gastric cancer [12], in LG group, 443 cases (21.7%) were at stage Ia, 155 cases (7.6%) at stage Ib, 218 cases (10.7%) at stage IIa, 237 cases (11.6%) at stage IIb, 216 cases (10.6%) at stage IIIa, 360 cases (17.6%) at stage IIIb, and 412 cases (20.2%) at stage IIIc. While, in the OG group, 227 cases (14.7%) were at stage Ia, 126 cases (8.2%) at stage Ib, 72 cases (4.7%) at stage IIa, 185 cases (12.0%) at stage IIb, 163 cases (10.6%) at stage IIIa, 264 cases (17.2%) at stage IIIb, and 502 cases (32.6%) at stage IIIc. There were more advanced gastric cancer in OG group than that in LG group (P<0.001).

**Table 1:** Clinicopathological Characteristics of patients (n = 3580).

**Characteristics**	**LG group (%, N = 2041)**	**OG group (%, N = 1539)**	**P value**
Age(years)	61.0±11.1	59.8±10.8	0.643
Gender			0.393
Male	1523 (74.6)	1170 (76.0)	
Female	518(25.4)	369 (24.0)	
Performed operation			0.159
Distal gastrectomy	938(46.0)	652 (42.4)	
Total gastrectomy	1049(51.4)	839 (54.5)	
Proximate gastrectomy	54(2.6)	48 (3.1)	
Histologic type			<0.0001
Differentiated	384(18.8)	397 (25.8)	
Undifferentiated	1657(81.2)	1142 (74.2)	
Tumor diameter (cm)	4.6±2.6	5.1±2.7	0.005
BMI（kg/m2）	22.3±3.2	22.6±3.5	0.734
Tumor location			0.087
Upper	613(30.1)	516(33.5)	
Middle	470(23.0)	338(22.0)	
Lower	958(46.9)	685(44.5)	
Depth of invasion			<0.0001
T1	516(25.3)	264(17.1)	
T2	234(11.5)	165(10.8)	
T3	558(27.3)	181(11.7)	
T4a	733(36.0)	929(60.4)	
pN stage			0.002
N0	779(38.2)	504(32.7)	
N1	287(14.1)	208(13.5)	
N2	351(17.2)	260(16.9)	
N3a	390(19.1)	355(23.1)	
N3b	234(11.5)	212(13.8)	
TNM stage			<0.0001
Ia	443(21.7)	227(14.7)	
Ib	155(7.6)	126(8.2)	
IIa	218(10.7)	72(4.7)	
IIb	237(11.6)	185(12.0)	
IIIa	216(10.6)	163(10.6)	
IIIb	360(17.6)	264(17.2)	
IIIc	412(20.2)	502(32.6)	
Chemotherapy			0.037
Yes	1527(74.8)	1246(81.0)	
No	514(25.2)	293(19.0)	
Vessel carcinoma embolus			0.367
Yes	582(28.5)	451(29.3)	
No	1459(71.5)	1088(71.7)	

### Perioperative and postoperative outcomes

A conversion to open laparotomy was required in 18 patients (0.9%). The reasons for conversion were uncontrolled diffuse bleeding in the operation field for 13 cases, abdominal adhesions for 3 patients, and peripheral viscera injuries in 2 patients (one with a transverse colon injury and the other with a spleen injury). There were no significant differences in volume of operation time (P = 0.399), time to first flatus (P = 0.526), and time to resumption of diet (P = 0.649) between the two groups. However, the blood loss (P<0.001), transfused patient number (P = 0.009), time to ground activities (P = 0.038), and post-operative hospital stay (P<0.001) was significantly less in the LG group than those in the OG group.

One or more complications occurred in 277 patients (13.6%) of LG group and 221 patients (14.4%) of OG group without statistical significant (P>0.05). Pulmonary problems were the most frequent in both two groups. Lymphorrhea, intraabdominal abscess, and wound infection were the most common problems in LG group with 38 (1.9%), 35 (1.7%), and 32 (1.6%) patients involved, respectively. While, wound infection, intraabdominal abscess, and lymphorrhea the most common problems in OG group. There were 6 patients (0.3%) in LG group who died by postoperative the 30^th^ day following the operation comprised 3 patients (0.2%) in OG group. The causes were anastomotic leakage and bleeding (3 patients), severe pneumonia (1 patient), disseminated intravascular coagulation (DIC) (1 patient), and infarct of spleen (1 patient) in LG group; in OG group there were two patients dead with anastomotic leakage and bleeding, one patient dead with pulmonary infection. (Table 2).

**Table 2:** Perioperative and postoperative results after laparoscopic and open gastrectomy.

	**LG group (%)N = 2041**	**OG group (%)N = 1539**	**P value**
Conversion to open surgery	18 (0.9%)	-	-
Operation time(min)	189.5±56.8	226.6±70.1	0.399
Blood loss(ml)	75.2±111.4	198.8±210.4	<0.0001
Transfused patients	14 (0.7%)	25(1.6%)	0.009
Time to ground activities (days)	2.3±0.7	3.0±0.9	0.038
Time to first flatus (days)	3.7±0.9	3.9±1.0	0.526
Time to resumption of diet (days)	4.8±1.1	4.7±1.2	0.649
Postoperative hospital stay (days)	13.8±6.8	15.1±8.3	0.013
Total complications	277 (13.6)	221(14.4)	0.526
Local complications	218 (10.7)	173(11.2)	0.626
Lymphorrhea	38 (1.9)	19(1.3)	
Intraabdominal abscess	35 (1.7)	28(1.8)	
Wound infection	32 (1.6)	29(1.9)	
Anastomotic leakage	24 (1.2)	17(1.1)	
Intestinal obstruction	22 (1.1)	18(1.2)	
Gastrasthenia	21 (1.0)	17(1.1)	
Abdominal bleeding	14 (0.7)	17(1.0)	
Anastomotic site bleeding	10 (0.5)	9(0.6)	
Pancreatic fistula	8 (0.4)	9(0.6)	
Duodenal stump leakage	8 (0.4)	5(0.3)	
Anastomotic straitly	5 (0.2)	5(0.3)	
Infarct of spleen	1 (0.05)	0	
Systemic complications	59 (2.9)	48(3.2)	0.693
Pneumonia	48 (2.4)	42(2.7)	
DIC	5 (0.2)	0	
Blood poisoning	4 (0.2)	5(0.3)	
Cerebral infarction	2 (0.1)	1(0.1)	
Postoperative mortality	6 (0.3)	3(0.2)	0.740

### Lymph node retrieval for LG and OG groups

The median number of retrieved LNs was 31 (range, 12–82; mean 31.1 ± 13.2) per patient. There was no significant difference in the total number of retrieved lymph nodes between the two groups (31.4±12.8 in the LG group vs 30.7±11.2 in the OG group; p = 0.445). According to the UICC TNM classification of gastric cancer, a comparative analysis of the total number of retrieved LNs showed no statistical significance for any of the stages of cancer, with the exception of stage IA, for which a more LNs was shown for the LG group. There were more positive LNs in OG group than that in LG group (6.6±8.5 vs. 5.7±8.2, P = 0.033). But there was no significant difference in the positive LNs at each stage between the two groups. (Table 3)

**Table 3:** Lymph node retrieval for LG and OG groups.

	**LG group**	**OG group**	**P value**
No. of retrieved LNs	31.4±12.8	30.7±11.2	0.445
Ia	27.8±12.5	25.1±10.3	0.003
Ib	29.9±13.3	29.0±11.7	0.626
IIa	32.4±12.9	30.7±11.9	0.539
IIb	31.2±11.3	30.1±10.3	0.165
IIIa	32.2±11.4	31.6±11.0	0.225
IIIb	34.1±13.8	33.3±12.6	0.378
IIIc	35.6±12.4	34.5±10.9	0.134
No. of positive LNs	5.7±8.2	6.6±8.5	0.033
Ia	0	0	-
Ib	0.4±0.6	0.3±0.6	0.434
IIa	0.7±1.2	0.7±1.0	0.923
IIb	1.9±2.5	1.3±2.3	0.256
IIIa	4.0±3.2	3.6±2.9	0.677
IIIb	9.6±7.9	9.1±6.6	0.514
IIIc	15.4±8.6	16.2±8.7	0.165

### Survival after the surgery

**Figure 1:**
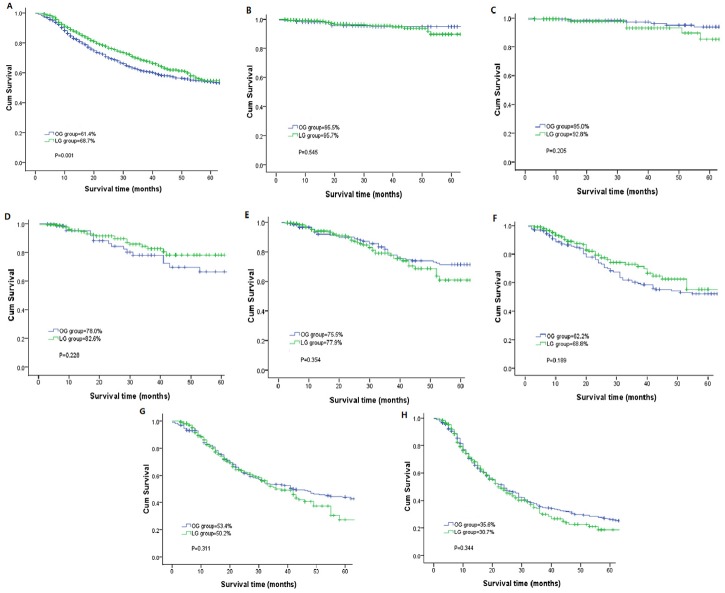
Kaplan-Meier curves for DFS. Kaplan-Meier curves for DFS between the laparoscopic gastrectomy and open gastrectomy groups.

**Figure 2:**
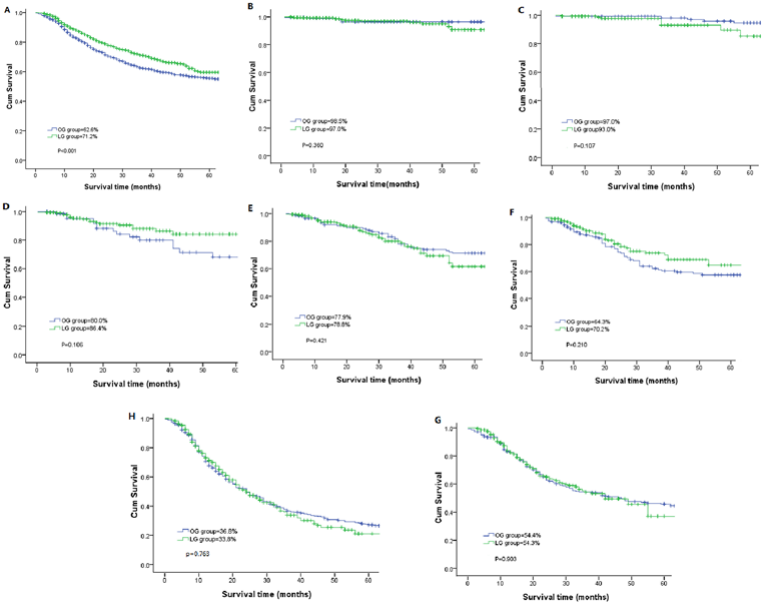
Kaplan-Meier curves for OS. Kaplan-Meier curves for OS between the laparoscopic gastrectomy and open gastrectomy groups.

During the follow-up stage, 81 patients in the LG group and 47 patients in the OG group were lost to follow-up. There were 456 patients in the laparoscopic group developed tumor recurrence, and the corresponding findings in the open group were 624 patients. The calculated 3-year disease-free survival (DFS) rate for all stages was 68.7% in the LG group and 61.4% in the OG group with significant difference (P<0.05). While the calculated 3-year DFS rate for patients in stage Ia was 95.7% in the LG and 95.5% in the OG; in stage Ib, 92.8% and 95.0%; in stage IIA, 82.6% and 78.0%; in stage IIB, 77.9% and 75.5%; in stage IIIA, 68.8% and 62.2%; in stage IIIB, 50.2% and 53.4%; and in stage IIIC, 30.7% and 35.6%, respectively. A comparative analysis of the DFS showed no statistical significance for any of the stages of cancer (Fig. 1). Similar findings were observed for the 3-year overall survival (OS) rates. The actual 3-year OS rate was 71.2% in the LG and 62.6% in the OG for patients, which was statistically significant (P<0.05). While the 3-year OS rate for stage IA was 97.0% in the LG group and 98.5% in the OG group; for stage IB, 93.0% and 97.0%; for stage IIA, 86.4% and 80.0%; for stage IIB, 78.8% and 77.9%; for stage IIIA, 70.2% and 64.3%; for stage IIIB, and 54.3% and 54.4%; for stage IIIC, 33.8% and 36.8%, respectively. Comparisons of the overall survival rates did not show statistical significance for any of the stages of cancer. (Table 4, Fig. 2).

**Table 4:** The calculated 3-year DFS rates and OS rates after laparoscopic and open gastrectomy.

**Stages**	**LG group (%)**	**OG group (%)**	**P value**
3-year DFS* rate	68.7	61.4	0.001
Ia	95.7	95.5	0.545
Ib	92.8	95.0	0.205
IIa	82.6	78.0	0.228
IIb	77.9	75.5	0.354
IIIa	68.8	62.2	0.189
IIIb	50.2	53.4	0.311
IIIc	30.7	35.6	0.344
3-year OS* rate	71.2	62.6	<0.0001
Ia	97.0	98.5	0.360
Ib	93.0	97.0	0.107
IIa	86.4	80.0	0.106
IIb	78.8	77.9	0.421
IIIa	70.2	64.3	0.210
IIIb	54.3	54.4	0.903
IIIc	33.8	36.8	0.763

*DFS: disease-free survival; OS: overall survival

## Discussion

Gastric cancer is one of the most common causes of cancer-related death in the world [12]. In most countries in the world, with exceptions including Japan and Korea, more than 80% of patients with gastric cancer are diagnosed with advance gastric cancer (AGC). Among the less invasive operations noted in recent years, laparoscopic gastrectomy for gastric cancer has become popular due to advances in surgical techniques. As experience with LG for early gastric cancer has accumulated, some centers have attempted to expand the indication of LG to AGC, and a few studies have already demonstrated that surgeons can safely perform laparoscopic gastrectomy in these cases with better qualities of life than those who underwent conventional open surgery [13–15]. But these retrospective studies of LG for AGC only included less advanced gastric cancer such as those at stage II or stage IIIa [16, 17]. The effects of LG for more advanced gastric cancer, such as stage IIIb and stage IIIc disease were rarely reported. In this study, we analyzed no only the early gastric cancer, but also some more advanced gastric cancer, and found that patients undergoing laparoscopic surgery had better postoperative recovery, with less blood loss, quicker intestinal functional recovery, and shorter hospital stay than those undergoing conventional open surgery too. Like the other laparoscopic procedures, there is a learning curve associated with laparoscopic gastrectomy, many surgeons are starting to perform this procedure with the tacit acceptance of a lengthy operation time because they often perceive LG to be a complicated technique inevitably subjected to the learning curve effect. A study by Kunisaki et al. [18] focused on one surgeon’s surgical learning curve with LADG showed that the operating time was reduced to 230 mins after 60 cases. Lee et al. [19] reviewed 257 patients who received distal gastrectomies (included 136 LADGs and 120 ODGs); they found that the mean operation times were similar between the two groups. Our team performed its first LAG for early gastric cancer in April 2007. After “climbing up” the learning curve, we found that the operation time gradually decreased. The average operation time was 237 min after 218 patients [20], and decreased to 196 min after we completed 1380 cases [21]. Now, the average operation time is only 189.5 min. A stable team with a tacit understanding plays an important role in laparoscopic gastrectomy, making the operation easier and faster.

The incidence of postoperative complications is still the most frequently used surrogate marker of the ‘‘quality’’ of surgery. Laparoscopic gastrectomy for gastric cancer has gained popularity. However, reported morbidity rates for laparoscopic surgery vary from 6.1 to 25.4% [22–25]. A Korean laparoscopic gastrointestinal surgical society (KLASS) trial [22], which is a multicenter, prospective, randomized clinical trial, included 179 laparoscopic-assisted and 163 open distal gastrectomy patients, reported an 11.6% early morbidity for the LAG and 15.1% for the OG, with a mortality of 1% for the LAG. Kitano et al. [23] published a multicenter trial with 1294 patients undergoing laparoscopic gastrectomy. The mortality and morbidity rates found were 0% and 14.8%, respectively, and the rate of conversion to open surgery was 1.1%. These studies show that laparoscopic gastrectomy requires smaller incision, is less painful, enables rapid recovery, and results in decreased or no differences in the incidence of postoperative morbidity and mortality compared with open surgery. In this study, the postoperative morbidity and mortality were 13.6% and 0.3% respectively, in the LG group and 14.4% and 0.2%, respectively, in the OG group, with no significant differences (P>0.05). Therefore, from these viewpoints, laparoscopic gastrectomy with extent lymph node dissection for gastric cancer is a safe and feasible choice.

Nowadays, more and more studies show that the procedure for gastrectomy with extensive lymph node dissection is well established and accepted as a standard practice for the treatment of AGC. So, besides the technical feasibility and favorable clinical outcomes of LG, the quality of lymphadenectomy is the most important factor in performing LG with extensive LN dissection. A Japanese study [25] found that adequate staging was possible for 86% of the patients who underwent LADG because more than 15 lymph nodes, the minimum requirement for tumor-node-metastasis staging, were retrieved. Song et al. [26] enrolled 75 patients who received standard D2 lymph nodes dissection (44 underwent LADG, and 31 underwent ODG), and found no significant differences in the total number of retrieved lymph nodes or node stations between the two groups. They suggested that LADG with D2 lymph node dissection is oncological compatible with OG. In the current study, as a way of comparing the oncology aspect of quality control between LG and OG groups, we compared the total number of retrieved lymph nodes, the positive LNs, and the number of LNs by their stages. Although, there was more positive LNs in LG group than that in OG group (P = 0.033), but this status disappeared when we compared the positive LNs by their stages. The results showed that there were comparable numbers of retrieved LNs between the LG group and OG group at each stage. The laparoscopic gastrectomy with extent lymph node dissection is technically possible, and the number of retrieved lymph node was sufficient for accurate staging.

The long-term oncologic result is very important to the use of laparoscopic gastrectomy. Nowadays, there are some multicenter, randomized controlled clinical trials, such as CLASS-01 by China laparoscopic gastrointestinal surgical society (CLASS), focused on the laparoscopic and open conventional surgery in the treatment of patients with local advanced gastric cancer. However, the confirmed results, including the oncologic outcomes, are still awaiting. Thus, before conducting a large multicenter phase III RCT comparing laparoscopic gastrectomy with open gastrectomy for AGC, it would be good to have the basis of a large retrospective study on the long-term outcomes for AGC after LG. Pak et al. [27] analyzed 714 consecutive patients who underwent LG for gastric cancer, and found that the 5-year overall survival rates were 96.4% in stage I, 83.1% in stage II, and 50.2% in stage III. Their results indicated that LG for gastric cancer had acceptable long-term oncologic outcomes. To date, oncologic outcomes after laparoscopic versus open gastrectomies for the treatment of AGC haves been reported in some studies [28–31]. Although oncologic safety seems to be identical between the groups, the sample size was relatively small and in some series the primary focus for analysis was on less advanced gastric cancers (some only included pT2 or pT3 patients). In the present study, we analyzed the surgical outcomes of a series of 2041 consecutive patients (74.7% patients with advanced gastric cancer) by laparoscopic group as compared with those treated using the open method. Although we showed that the long-term oncologic outcome was better for LG group than that in OG group. This survival benefit of laparoscopic group probably arose from heterogeneity of two groups. A comparison of the clinical factors between the two groups showed that lager tumor diameter, and more advanced cancers were dominant in patients who underwent open conventional surgery. After comparing the survival rates according to each stage, this benefit of survival disappeared. So our result showed that the long-term oncologic results showed that the laparoscopic approach was not statistically inferior to the open conventional approach for the treatment of gastric cancer, even in patients with advanced gastric cancer. Although the present study still has limitations due to the nature of our consecutive but retrospective study design and may have been biased by the patient selection criteria, the current data support the idea that laparoscopic gastrectomy is an oncologically safe treatment for advanced gastric cancer.

In conclusion, the surgical outcomes of LG for gastric cancer including AGC in the present study seem to be comparable to those previously reported. However, further randomized trials will provide valuable evidence for the oncological safety of laparoscopic gastrectomy for treatment of advanced gastric cancer.
